# Eating disorder symptoms and control‐seeking behavior

**DOI:** 10.1002/brb3.3105

**Published:** 2023-06-28

**Authors:** Ashley Slanina‐Davies, Oliver J. Robinson, Alexandra C. Pike

**Affiliations:** ^1^ Anxiety Lab, Neuroscience and Mental Health Group, Institute of Cognitive Neuroscience University College London London UK; ^2^ Department of Psychology and York Biomedical Research Institute University of York York UK

**Keywords:** behavioral task, control‐seeking, eating disorders, intolerance of uncertainty, online

## Abstract

**Objective:**

Eating disorders (EDs) are a heterogenous group of disorders characterized by disturbed eating patterns. Links have been made between ED symptoms and control‐seeking behaviors, which may cause relief from distress. However, whether direct behavioral measures of control‐seeking behavior correlate with ED symptoms has not been directly tested. Additionally, existing paradigms may conflate control‐seeking behavior with uncertainty‐reducing behavior.

**Method:**

A general population sample of 183 participants completed part in an online behavioral task, in which participants rolled a die in order to obtain/avoid a set of numbers. Prior to each roll, participants could choose to change arbitrary features of the task (such as the color of their die) or view additional information (such as the current trial number). Selecting these Control Options could cost participants points or not (Cost/No‐Cost conditions). Each participant completed all four conditions, each with 15 trials, followed by a series of questionnaires, including the Eating Attitudes Test‐26 (EAT‐26), the Intolerance of Uncertainty Scale, and the Obsessive–Compulsive Inventory—Revised (OCI‐R).

**Results:**

A Spearman's rank test indicated no significant correlation between total EAT‐26 score and total number of Control Options selected, with only elevated scores on a measure of obsessions and compulsivity (OCI‐R) correlating with the total number of Control Options selected (*r*_s_ = .155, *p* = .036).

**Discussion:**

In our novel paradigm, we find no relationship between EAT‐26 score and control‐seeking. However, we do find some evidence that this behavior may be present in other disorders that often coincide with ED diagnosis, which may indicate that transdiagnostic factors such as compulsivity are important to control‐seeking.

## Graphical abstract

Links have been made between eating disorder symptoms and control‐seeking behaviors, which may cause relief from distress. In this study, we designed a novel behavioral task that allowed us to quantify control‐seeking behavior where this behavior was orthogonal to the task goals. We found no relationship between eating disorder symptoms and control‐seeking, though we did find evidence of a correlation between a measure of obsessions and compulsions and control‐seeking, which may hint to a role of compulsivity in control‐seeking.

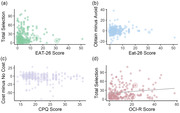

## 1 INTRODUCTION

Eating disorders (EDs) are a heterogeneous group of neuropsychiatric disorders with symptomology characterized by disturbed eating patterns. EDs include anorexia nervosa, binge eating disorder, bulimia nervosa, and other specified feeding or eating disorder. The mortality rates of EDs are among the highest of any mental health disorder (Arcelus, 2011; van Hoeken & Hoek, 2020), with a clinical or subclinical morbidity of over 50% (Steinhausen, 2002).

The notion of “control” or “personal control” has been frequently associated with EDs (Barca & Pezzulo, 2020; Froreich et al., 2016; Polivy & Herman, 2002; Sarra & Abar, 2022). Indeed, one of the major components of the transdiagnostic cognitive behavioral therapy formulation is phrased in terms of “control” over weight and shape (Murphy et al., 2010). Control‐seeking may, however, not purely be limited to weight and shape: those with EDs may have an elevated need to control their thoughts (Palmieri et al., 2021), and they may also experience less of a sense of control over the external world (Dalgleish et al., 2001). The exploration of control‐seeking as a trait in mental illnesses has often involved using paradigms where the participant is able to reduce uncertainty by engaging with the task (Jacoby et al., 2014; Sternheim, Startup et al., 2011). The beads task is one such example: here, participants remove beads from an urn until they feel able to state the most prevalent color of beads within the urn (Huq et al., 1988). Crucially, in scenarios such as this, the more beads a participant selects, the more uncertainty is reduced, making it impossible to understand whether the primary motivation in selecting more beads is uncertainty reduction or control‐seeking.

Differentiating between control‐seeking and uncertainty reduction is likely to be important, as recent work has suggested that in the ED population, control‐seeking might be a response to elevated intolerance of uncertainty (IU), defined as the “desire for predictability and an active engagement in seeking certainty” with a “paralysis of cognition and action in the face of uncertainty” (Birrell et al., 2011). IU is elevated across different ED categories (Brown et al., 2017), and evidence suggests it may have a clinically important role in ED development, maintenance, and/or recovery (Kesby et al., 2017). Specifically, a focus group study conducted by Sternheim et al. (2011) related IU in EDs to a need or desire for control: in the face of uncertainty, the distress experienced is such that the individual feels compelled to gain a sense of certainty by controlling their immediate environment. In the aforementioned study, this was described as taking the form of avoidance, routine‐seeking, and excessive planning—with the ultimate focus of control being over food and weight.

An additional complication in understanding the role control‐seeking may play in EDs is that multiple different cognitive biases or processes may interact or conflict with control‐seeking behavior when individuals select a behavioral strategy. For example, increased avoidance of both one's own body (Nikodijevic et al., 2018) and internal cognitions and emotions in general (Rawal et al., 2010) has been implicated across EDs, and so it may be that avoiding negative outcomes is more salient than obtaining positive outcomes (Harrison et al., 2011), which may impact the situations in which control‐seeking becomes apparent. Similarly, perfectionism has been implicated across EDs (Shafran et al., 2002), which may act to reduce control‐seeking behavior if a goal conflicts with control‐seeking. This makes both avoidance and perfectionism key features to model when attempting to understand any relationship between EDs and control‐seeking.

As such, in this study we designed a novel behavioral task to elicit arbitrary control‐seeking behaviors and test whether task performance was related to disordered eating attitudes and other relevant mental health symptoms.

## 2 METHODS

### 2.1 Procedure

The experiment comprised an online behavioral task followed by a series of self‐report questionnaires delivered via the host website Gorilla.sc (Anwyl‐Irvine et al., 2020). Participants were an online convenience sample of Prolific.ac users (Palan & Schitter, 2018) and were remunerated at a rate of £7.50 per hour. All participants provided informed consent, in line with UCL Ethics approval 15253/001. The experiment was preregistered on the Open Science Framework: https://doi.org/10.17605/OSF.IO/SZ8FD.

### 2.2 Participants

Participants were included if they were aged between 18 and 100 years old, had normal or corrected to normal vision, were fluent in English, and had no history of cognitive impairment or dementia. We recruited 183 participants, to allow us to achieve 95% power using a one‐tailed random effects multiple regression model for a minimally interesting effect size of *ρ* ^2^ = .1 (Faul et al., 2007), given an expected 10% participant exclusion rate.

### 2.3 Task

**FIGURE 1:**
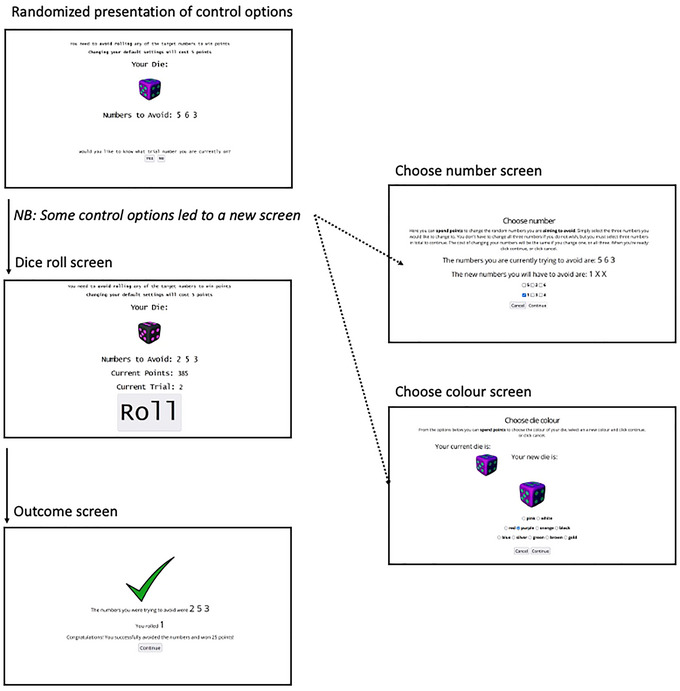
Task trials. Participants start on the main page (top left), with a random die and three random target numbers. They are asked a series of five questions, which appear in a random order for each trial, regarding changes they can make (the Control Options): “Yes” or “No” must be answered to progress. The top right panel shows the page for changing the target numbers (the target number options appear in a random order), and the bottom right shows changing the die color (all color options appear in a random order). The middle page on the left shows the main page after the selection of additional information (showing current points and trial number). See Supporting Information for more information regarding the Control Option categories. The bottom left is the feedback screen—which would show a red cross or green tick, depending on if the task conditions have been met. The die was not shown on the “Rolling” or “Feedback” screen to minimize any association between colors and outcomes. After the feedback screen, participants return to the top left to begin a new trial with a new randomly assigned die and numbers, repeating this cycle until all four blocks had been completed: Avoid/No‐Cost; Avoid/Cost; Obtain/No‐Cost; and Obtain/Cost. Importantly, participants were told that all dice were fair, that they did not need to select any Control Options to continue, and the monetary reward would be at a fixed rate irrespective of any points won or spent. These instructions highlighted the arbitrary nature of all Control Options presented.

The task had a 2 × 2 repeated‐measures design (with the two factors being Framing and Cost), and consisted of 60 trials split evenly into the resulting four conditions. On each trial, participants were shown one of 99 random dies varying according to the color of the faces (11 options) and the color of the dots (nine options), along with three random numbers and instructed to either roll, or avoid rolling, one of these numbers (Obtain/Avoid framing) to win 25 points. Participants were then presented with five questions, in a random order, regarding changes they could make to the task space prior to rolling the die (the five Control Options), and told there would either be a cost or no cost for selecting a change (Cost/No‐Cost conditions). For the purposes of this explanation, one can conceive of the Control Options as falling into three categories. The first category allowed participants to display information regarding the trial number and current points total, the second category allowed participants to change the target numbers, and the third category allowed participants to change the color of the die they would roll during the trial. If a participant selected to display information, the information was immediately added to the screen for the duration of the trial. If a participant selected to change the target numbers or colors, they were navigated to a screen showing the alternative options, before making their selection and returning to the main task screen. Importantly, participants were told that all dice were fair, that they did not need to select any Control Options to continue, and the monetary reward would be at a fixed rate irrespective of any points won or spent. These instructions highlighted the arbitrary nature of all Control Options presented. The task is depicted in Figure 1.

### 2.4 Questionnaires

Participants completed eight self‐report questionnaires: to capture the main construct of interest (eating disorder symptoms), we used the Eating Attitudes Test 26 (EAT‐26: Garner et al., 1982), with the omission of Part A (questions regarding weight and height) as these questions do not contribute to total scores, and addition of a single question at the end of the self‐report to screen for potential confounds in scoring results: “Do you feel your answers have been affected by factors other than your attitude toward food and body shape? Such as by having a food allergy or physical illness?,” with response options of “Yes” and “No”; complementary measures of IU (Intolerance of Uncertainty Scale [IUS: Freeston et al., 1994]), depression (Patient Health Questionnaire 8 [PHQ‐8: Kroenke et al., 2009]), generalized anxiety (Generalized Anxiety Disorder seven‐item scale [GAD‐7: Spitzer et al., 2006]), impulsivity (Barratt Impulsiveness Scale [BIS: Patton, 1995]), compulsivity (Obsessive–Compulsive Inventory Revised [OCI‐R: Foa et al., 2002]), perfectionism (Clinical Perfectionism Questionnaire [CPQ: Egan et al., 2016; Fairburn et al., 2003]), and self‐esteem (Rosenberg Self‐Esteem Scale [RSES: Rosenberg, 1965]) were also taken. Finally, we collected information regarding any mental health diagnosis and medication, along with questions on the impact of the COVID‐19 pandemic.

All questionnaires were delivered after the main task in a pseudorandomized order (see preregistration for further details).

### 2.5 Hypotheses

#### 2.5.1 H1: Those with disordered eating will engage in more control‐seeking behavior

Specifically, we predicted higher scores on the EAT‐26 would correlate positively with instances of arbitrarily manipulating the experimental environment as measured by sum total of Control Option selection.

#### 2.5.2 H2: Avoid conditions will increase instances of control‐seeking behavior in those with disordered eating, when compared to Obtain conditions

Specifically, we predicted there would be a negative correlation between EAT‐26 scores and the difference in total Control Options selected for Obtain minus Avoid conditions, based on the prediction that avoidance may be particularly relevant to EDs.

#### 2.5.3 H3: Instances of control‐seeking will correlate negatively with perfectionism in Cost conditions

We predicted there would be a negative correlation between Clinical Perfectionism Questionnaire scores and the difference in total Control Options selected for Cost minus No‐Cost conditions, based on the hypothesis that perfectionism will interact with control‐seeking.

### 2.6 Analysis

All data were analyzed in line with the preregistration using R statistical software version 4.0.2. Analysis was performed regardless of participants’ response to the EAT‐26 confound question. However, we also performed a sensitivity analysis excluding those who responded “yes” to this question. We also performed a mediation analysis (using the “mediation” package in R v.4.5.0, with 1000 simulations and nonparametric bootstrap confidence intervals using the percentile method) to assess whether IU mediated the relationship between EAT‐26 scores and control option selection.

### 2.7 Preregistered exploratory analysis

As per the preregistration, we also performed a number of additional preregistered exploratory analyses. These additional analyses sought to explore: whether the selection of Control Options from the different categories was related to any particular mental health measure (H4); how Control Option selection varied with time, both in terms of the number of Control Options selected, that is, perseverance (H5), and the specific options selected, that is, rigidity versus flexibility in exploring the task space (H6). Further information and the results can be found in the Supporting Information.

## 3 RESULTS

A total of 183 participants completed the study (106 female; for demographics, see Table 1). Ten participants indicated their answers to the EAT‐26 questionnaire were influenced by factors other than their attitude to food and body image. Where exclusion of these participants had any effect on the significance of results, this is indicated and both results reported below, otherwise the results reported are for the complete dataset.

**TABLE 1:** Demographic details, questionnaire scores, and task details for this study.

Measure	Median	IQR
Prolific data		
Age	44	34−53
Time taken (min)	34	29−42
Prolific score (/100)	100	99−100

Questionnaire scores		
EAT‐26 score	4	2−8
PHQ‐8 score	5	1−8
GAD‐7 score	4	1−8
IUS score	58	43.6−76.5
BIS‐11 score	57	51−63
OCIR score	10	4−19
CPQ score	21	18−26
RSES score	19	13−23

Control Option selection		
Total Control Options	16	7−34
Avoid framing	8	3−17
Obtain framing	9	2.5−18
Cost condition	7	1−15
No‐Cost condition	9	3−18

*Note*: We show the median and interquartile range of participants’ ages, the time they took to complete the entire protocol (informed consent, task, and questionnaires), their Prolific score (out of 100; this indicates how many good quality submissions participants have made—high scores indicate well‐performing participants who generally attend to tasks and follow instructions), their scores on the self‐report questionnaires (EAT‐26—Eating Attitudes Test, 26 item version; PHQ‐8—Patient Health Questionnaire depression scale, eight‐item version; GAD‐7—Generalized Anxiety Disorder seven‐item scale; IUS—Intolerance of Uncertainty Scale; BIS‐11—Barratt Impulsiveness Scale; OCI‐R—Obsessive–Compulsive Inventory Revised; CPQ—Clinical Perfectionism Questionnaire; RSES—Rosenberg Self‐Esteem Scale), and the number of control options they selected in different conditions. Data are for all 183 participants, of whom 103 were female.

Abbreviation: IQR, interquartile range.

### 3.1 Characterizing the control task

**FIGURE 2:**
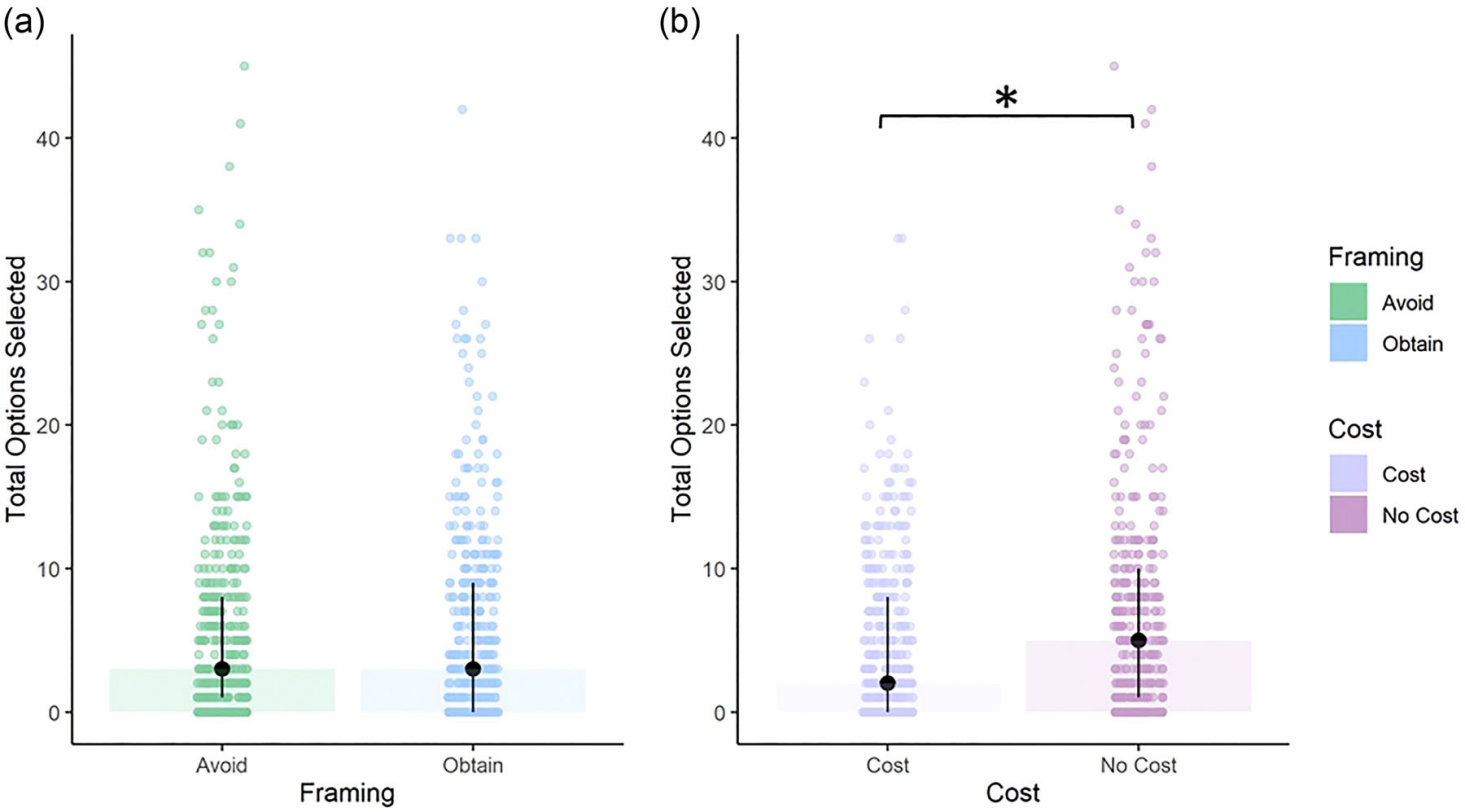
Effects of Condition and Position. Participants completed the die‐rolling task. On each trial, prior to rolling the dice, they could select various options (“Control Options”) that might allow them to gain information about their points total, change the visual appearance of the dice, or change the target numbers. The task had four different conditions: two separate framings (either participants were trying to avoid or obtain particular target numbers on dice rolls) and two different cost conditions (selecting control options either cost points or did not). In a two‐way repeated‐measures ANOVA, there was (a) no main effect of framing on the number of control options selected (*F*_(1, 182)_ = 0.00, *p* = .99), but there was (b) a main effect of Cost (*F*_(1, 182)_ = 25.53, *p* < .01). There was no interaction between Framing and Cost (*F*_(1, 182)_ = 3.55, *p* = .061). Left axis always shows total number of Control Options selected, dot plots show data points for all participants, and bar chart is drawn at the median. *p < .05.

In a repeated‐measures ANOVA including Cost (Cost vs. No‐Cost block) and framing (Avoid vs. Obtain), there was no main effect of Framing on the total number of Control Options selected (*F*_(1, 182)_ = 0.00, *p* = .99; Figure 2a), but there was a significant main effect of Cost on the total number of Control Options selected (*F*_(1, 182)_ = 25.53, *p* < .01; Figure 2b). There was also no interaction between Cost and Framing (*F*_(1, 182)_ = 3.55, *p* = .061).

### 3.2 Hypothesis testing

#### 3.2.1 H1: No relationship between ED symptoms and Control Option selection

**FIGURE 3:**
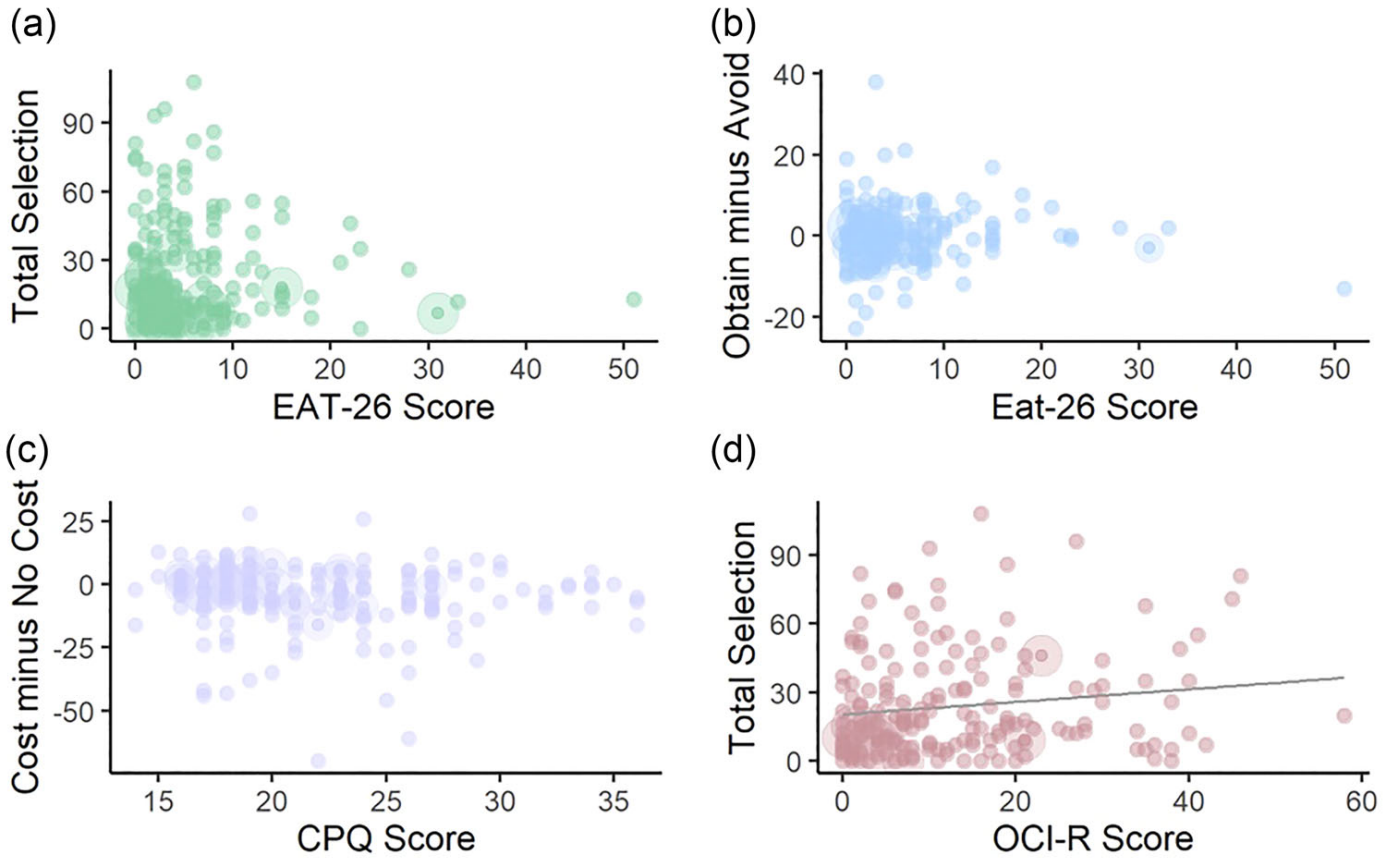
H1–H3: (a) There was no correlation between EAT‐26 (*r*_s_ = .12, *p* = .87) and the total number of Control Options selected and (b) no correlation between Obtain–Avoid Control Option selection and EAT‐26 score (*r*_s_ = .061, *p* = .41). (c) There was no relationship between Cost minus No‐Cost Control Option selection and CPQ score (*r*_s_ = −.14, *p* = .054), though note that this was significant after the exclusion of those who responded that a confounding factor could have influenced their responses to the EAT‐26 (*r*_s_ = −.16, *p* = .034). (d) There was a significant positive relationship between OCI‐R scores and total Control Option selection, though this did not survive correction for multiple comparisons (*r*_s_ = .155, *p* = .036).

**FIGURE 4:**
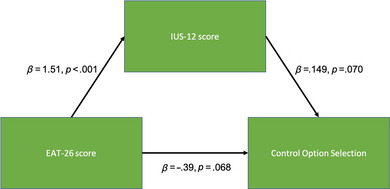
A path diagram showing a mediation analysis. We were interested in whether a potential relationship between EAT‐26 scores and control option selection was mediated by responses to the Intolerance of Uncertainty questionnaire. The overall effect was not significant, limiting any possible interpretation, although the relationship between the EAT‐26 and IUS‐12 scores was significant, as was the average causal mediation effect. Green boxes represent measured variables, and arrows show the direction of regressions, with annotations indicating the estimated size of effects and significance.

Contrary to our hypothesis, we found no correlation between EAT‐26 score and Control Option selection (Spearman's rank *r*_s_ = .12, *p* = .87) (Figure 3a). An analysis to examine whether IU was a mediator between EAT‐26 score and Control Option selection was inconclusive, as the total effect was not significant (*β* = −0.168 [−0.464, 0.210], *p* = .344). Notably, although the average direct effect was also not significant (*β* = −0.394 [−0.821, 0.030], *p* = .068), the average causal mediation effect was significant (*β* = −0.226 [0.010, 0.480], *p* = .044). This relationship is illustrated in Figure 4.

#### 3.2.2 H2: No relationship between the effect of “avoid” condition on Control Option selection and ED symptoms

We also predicted increased instances of Control Option selection in Avoid conditions in those with higher EAT‐26 scores. To calculate the dependent variable of interest, the total number of Control Options selected in Avoid conditions was subtracted from the total number of Control Options selected in Obtain conditions. There was no significant correlation between this and EAT‐26 scores (*r*_s_ = .062, *p* = .41) (see Figure 3b).

#### 3.2.3 H3: No relationship between the effect of “cost” condition on Control Option selection and self‐reported perfectionism

We found no significant relationship between the difference in Control Options selected in Cost versus No‐Cost conditions and CPQ score (*r*_s_ = −.14, *p* = .054; Figure 3c).

#### 3.2.4 Sensitivity analyses

The results of H1 and H2 did not differ when excluding participants who answered that there was a factor that may confound their scores on the EAT‐26 questionnaire. However, when these participants were excluded from the analysis of H3, the negative correlation between the two measures reached significance (*r*_s_ = −.16, *p* = .034, uncorrected).

#### 3.2.5 Exploratory analysis

In an un‐preregistered exploratory multiple regression, we examined whether any questionnaire score had a relationship with Control Option selection. Notably, there was no relationship between IUS score and number of Control Options selected (*β* = 0.059 [*SE* = 0.123], *p* = .633). The highest regression estimate was for OCI‐R score (*β* = 0.374 [*SE* = 0.210], *p* = .077). In a Spearman's rank correlation test, this is significant (*r*_s_ = .155, *p* = .036) (Figure 3d). Notably, if we performed a correlation test for all questionnaires against total option selection, this finding would not survive correction for multiple comparisons (seven tests, making Bonferroni adjusted alpha = .007).

## 4 DISCUSSION

In this study, we developed a behavioral task to investigate control‐seeking as a response to uncertainty. In particular, the metric we argue reflects control‐seeking—selecting more “Control Options”—is of no utility: uncertainty is not reduced and no task‐relevant information is gained by selecting Control Options. We used this task to investigate whether control‐seeking is related to ED symptoms: counter to our main hypothesis (H1), we found no evidence of a correlation between Control Option selection and our measure of disordered eating (EAT‐26). Also contrary to our second hypothesis (H2), we did not find a relationship between EAT‐26 scores and enhanced Control Option selection in an “avoidance” framing, neither did we find robust evidence of perfectionism associating with reduced Control Option selection in Cost conditions, where point loss was diametrically opposed to Control Option selection (H3).

### 4.1 Task characterization

We were able to show that adding a cost for selecting Control Options reduced the number of options selected, though Avoid/Obtain framing had no effect.

### 4.2 H1: No relationship between control‐seeking and ED symptoms

In contrast to our prediction, we found no evidence of a correlation between EAT‐26 score and total Control Option selection in our sample of the general population. This may suggest that control‐seeking (when measured independently from uncertainty reduction) does not increase with increasing levels of ED pathology in a nonclinical population. In operationalizing this proposed relationship, we looked specifically at arbitrary control‐seeking in response to short‐term, low‐stress uncertainty. As such, our results could indicate that control‐seeking does not occur outside of specific cases where control‐seeking also adds information/reduces uncertainty or that such behaviors might not be apparent in nonstressful or emotive environments (e.g., online tasks compared to high uncertainty/stress situations). While further evidence is needed to show that uncertainty reduction is a main aim of control‐seeking behaviors in EDs, if this is corroborated, there may be clinical utility to focusing on uncertainty and tolerating uncertainty rather than sense of or need for control per se. This would perhaps treat *both* distress around uncertainty and perceived “control‐seeking” behaviors. However, it is worth noting the limitations below when interpreting this result, and also that we were only powered to detect an effect of a certain size. There may be an association between control‐seeking and ED symptoms that is smaller than we considered meaningful in our power analysis. Furthermore, noise or inadequate psychometric properties of our measures may have limited our ability to detect a significant result: our task may not have adequately operationalized control‐seeking, or perhaps the EAT‐26, as a clinical screening tool, is not appropriate for use in the general population in a correlational analysis.

### 4.3 Relationship between control‐seeking and obsessive–compulsive symptoms

We did observe a potential relationship between OCI‐R scores and Control Option selection, though this would not survive correction for multiple comparisons. If this finding was replicated in future work, it would be consistent with prior work showing increasing trait compulsivity is associated with increasing information‐gathering behaviors across the clinical and nonclinical spectrum (Hauser et al., 2017). This might lead to the interpretation that the increased selection of Control Options in this task is not an attempt to exert arbitrary control over the task space, but an attempt to explore and confirm the task dynamics. It would be interesting to see whether there are circumstances under which these behaviors might extend to those with EDs, given their relationship with compulsivity (Godier & Park, 2016) and the clinical emphasis on control in both disorders.

### 4.4 H2: No relationship between our “avoidance” manipulation and ED symptoms

By using different framings—Avoid and Obtain—we had hoped to probe the propensity to engage in control‐seeking behavior based on the desire to avoid harmful/negative outcomes. This was based on previous findings that uncertainty can be perceived as harmful (Frank et al., 2012), and so control behaviors increase as an attempt to avoid harm. There was no such relationship in terms of preferential Control Option selection in Avoid conditions. This might suggest that avoidance of outcomes does not motivate control‐seeking in those with ED symptoms. Alternatively, given that we did not identify an effect of framing on the whole participant group, our “avoidance” manipulation may have been unsuccessful.

### 4.5 H3: No relationship between our “cost” manipulation and self‐reported perfectionism

We did not find a relationship between self‐reported perfectionism scores and tendency to select fewer Control Options in the “Cost” condition. However, in a sensitivity analysis in which we removed participants who expressed that their responses to the EAT‐26 questionnaire may have been confounded by other factors such as dietary or health issues, there is a significant relationship. We conclude that there is a general tendency for all participants to select less Control Options when they incur a cost, and this effect may be slightly more pronounced in those who have high perfectionist traits, but it is difficult to draw any firm conclusions from the data given the inconsistency in results. If the result is not an artifact, those with higher perfectionism may be more likely to pursue the “certain” goal of the preservation of points. Here, perfectionism may “beat” control‐seeking, or indeed, one could conceptualize perfectionism in terms of task performance as a form of control. It is possible that seeking better task performance is a more concrete goal than control‐seeking, and therefore “wins” out.

### 4.6 Limitations

Both the EAT‐26 scores and the number of Control Options selected are skewed toward zero (see Table 1). While Control Option selection was designed to be a minimally motivated action and so the low incidence of selection was somewhat expected, the exaggerated skew in *both* variables reduces the sensitivity of our analysis. This is because it is hard to identify a correlation when both distributions are very narrow: all else being equal, the value of a correlation will be greater if there is more variability among the relevant variables. This phenomenon is sometimes known as range restriction (Goodwin & Leech, 2006). Additionally, we use a correlational approach to assess control‐seeking, but it is possible that control‐seeking is not a linear function of symptoms and only emerges with more severe pathology. Participants also selected fewer Control Options as the experiment progressed (see Supporting Information), perhaps indicating fatigue, which could be improved in future designs. As we used a single fixed level of uncertainty, we also cannot infer that control‐seeking is directly related to uncertainty, and indeed, there was no correlation between IU and Control Option selection. Since dice were used to implement uncertainty, it would be simple to vary uncertainty in a future experiment by, for example, changing the quantity of target numbers, or the number of dice per trial, and directly compare results. Additionally, we did not explore the divergent or convergent validity of the task by comparison with other tasks or questionnaires, such as the beads task. Future research could use both our task and the beads task in the same population, in order to directly examine whether uncertainty reduction is a necessary component of control‐seeking behavior. We also did not take measures of cognitive flexibility or self‐control, which may influence behavior. Our task operationalizes “control‐seeking” as a preference for dictating the experimental environment, via engaging in arbitrary task changes that do not impact actual uncertainty about the task or provide any other benefit to task performance. However, this behavior could also be explained by other behaviors, including information‐seeking (in the form of checking or exploring the task space). Please see the Supporting Information for a discussion on the different Control Option categories, which may speak to some of these alternative explanations.

## 5 CONCLUSION

In contrast to our primary hypothesis, we found no evidence of a correlation between arbitrary control‐seeking and disordered eating (H1); this was not influenced by attempting to avoid outcomes (H2), nor was perfectionism related to the trade‐off between point deduction and control‐seeking (H3). There was a weak exploratory relationship between OCD symptoms and control‐seeking behavior, but this warrants replication. This research will help us to understand who may be vulnerable to different behaviors and thus better target treatments.

## CONFLICT OF INTEREST STATEMENT

O.J.R.’s MRC senior fellowship is partially in collaboration with Cambridge Cognition (who plan to provide in‐kind contribution) and he is running an investigator‐initiated trial with medication donated by Lundbeck (escitalopram and placebo, no financial contribution). He also holds an MRC‐Proximity to discovery award with Roche (who provide in‐kind contributions and have sponsored travel for ACP) regarding work on heart‐rate variability and anxiety. He has also completed consultancy work on affective bias modification for Peak, online CBT for IESO digital health, and on neural mechanisms of anxiety for Roche and Blackthorn therapeutics. O.J.R. previously sat on the committee of the British Association of Psychopharmacology. A.C.P. has received funding from the Wellcome Trust (Grant ref: 226694/Z/22/Z). A.S.‐D. declares no conflicts of interest.

### PEER REVIEW

The peer review history for this article is available at https://publons.com/publon/10.1002/brb3.3105

## Supporting information

Supplementary Materials

## Data Availability

All data, materials, and code, alongside the preregistration for this study, are available at https://osf.io/r5f83/?view_only=163d208ce5b54642b6bc496839974868.
