# Supplementary material for: Eating disorder symptoms and control‐seeking behavior
Source: Brain Behav. 2023 Jun 28;13(8):e3105. doi: 10.1002/brb3.3105 (PMC10454257; doi:10.1002/brb3.3105)
Supplement: Supplementary file 1 — Supplementary Materials [file BRB3-13-e3105-s001.docx]

Supplementary Materials

# Supplementary Methods

## Deviations from pre-registration

Due to the non-normal distribution of residuals, we deviated from the original plan of analysis and used Spearman’s rank correlation for hypothesis testing. Unless otherwise stated, this statistical test was used to obtain the results reported in the main paper.

Structural equation modelling using lavaan v0.6-9 was also performed to enable us to test associations whilst allowing for measurement error. In particular, we used this approach to further expand on the mediation analysis reported in the main text, and test whether IU might mediate any relationship between EAT-26 scores and Control Option selection. We used a structural equation model in which each questionnaire loaded onto its own unique latent factor, and then modified the resulting regressions that we allowed in line with our hypotheses. In this model, we regressed difference between Control Option selections between Cost/No-cost conditions against the latent factor of perfectionism, and the difference between Avoid/Obtain condition Control Option selection against the latent factor of eating disorders. We compared one structural equation model in which intolerance of uncertainty mediated the relationship between the eating disorder factor and number of Control Options selected, and one in which the relationship was direct, using an ANOVA. We report standardized coefficients where appropriate. These analyses were not pre-planned. Note that this SEM may be underpowered so should be treated with caution.

## Control Option sub-types

We designed the task such that we could examine different features of control-seeking behaviour. The Control Options participants could choose between represented three different ‘styles’ of control-seeking:

### Information Control Options:

The questions: “*Would you like to display your current trial number?*” and “*Would you like to display your current points total?*” might be more attractive to those seeking more information about the task, and were thus designated Information Control Options.

### Pseudo-Instrumental Control Option:

The question “*Before you roll, would you like to change [your target numbers]/[the numbers you are trying to avoid]?*” allowed participants to interact with the parameters which more closely influenced the outcome of the trial – though note that as the roll was independent of the numbers chosen, the probability of success was not altered. This option was designated a Pseudo-Instrumental Control Option, and allowed us to assess whether options that provided more apparent instrumental control were more valued.

### Aesthetic Control Options:

Finally, the questions “*Before you roll, would you like to change the colour of your die?*” and “*Before you roll, would you like to change the colour of the dots on your die?*” were designated Aesthetic Control Options. These options were most closely related to the experimental question of whether IU promotes arbitrary control-seeking in those with higher EAT-26 scores, as they were unrelated to either gaining information about the task or controlling the goal of the task.

# Pre-registered exploratory hypotheses

## H4: Exploratory analysis regarding Control Option category preference.

The Information Control Options gave participants additional, but outcome-irrelevant, information, which we used to explore whether information gathering is a strategy pursued even when information does not reduce outcome uncertainty and is not beneficial to task performance. We hypothesised that such a strategy might be related to compulsivity, given the previously reported relationship between compulsive traits and increased information gathering (NSPN Consortium et al., 2017). This question is of interest here given that compulsive behaviours are features of ED symptoms (e.g. checking calorific content and rigidly adhering to an exercise routine). We also hypothesised that this type of control option might be related to IU. We also explored which participants selected the Pseudo-instrumental Control Option, and Aesthetic Control Option selection (the category most closely related to the question of whether arbitrary control has greater intrinsic value to those who suffer from disordered eating).

## H5: Exploratory analysis regarding change in Control Option selection over time.

In total, the experimental task-set contains 60 trials in 4 different conditions. We were interested in understanding how Control Option selection may vary over time, and how this may correlate to different trait dimensions, particularly compulsivity (as measured by OCI-R score). As perseverative behaviours can characterise compulsivity (Luigjes et al., 2019), we predicted that there would be a negative correlation between time (block number) and sum total Control Option selection, and this would interact with OCI-R. We also performed an ANOVA to examine whether, in general, there was an effect of time on the task, by assessing whether block number was associated with the number of Control Options selected.

## H6: Exploratory analysis regarding Control Option selection flexibility vs rigidity.

Within the Aesthetic and Pseudo-Instrumental Control Options, there was the opportunity to explore the task space (i.e. alternatively choose different options) or to pursue a fixed strategy (repeatedly select a consistent set of Control Options). In psychopathology these two approaches may be associated with impulsive and compulsive traits (respectively). Both of these traits are thought to be elevated across EDs, so we wish to understand if in situations of uncertainty they influence response styles, leading to either behavioural flexibility or rigidity option selections. To explore this, we calculated repeat-selections divided by total-selections and tested any correlation this value might have against BIS and OCI-R score, expecting a negative and positive correlation (respectively).

# Results

## H4: Control Option category preference

To test any preference for Control Option type, the total number of Control Options selected was summed within the three categories of Information, Pseudo-Instrumental, and Aesthetic Control Option for each participant. We examined whether Information Control option selection was related to OCI-R scores or IU scores in a multiple regression, but this was not the case (all *p*>0.1). When all questionnaire scores were entered into a multiple regression, no questionnaire was significantly related to total option selection (all *p*>0.1). In a similar multiple regression including all questionnaire scores with the dependent variable of Pseudo-Instrumental Control Options, there was no indication of a correlation with any of the self-report measures (all *p*>0.1). However, Aesthetic Control Option selection was related significantly to OCI-R scores in a multiple regression including all questionnaires (*β* = 0.22, *p* = 0.006). Overall, participants chose significantly more Information Control Options than Control Options belonging to either of the other two categories (single factor ANOVA within subject Control Option category (three levels: Information; Pseudo-Instrumental; Aesthetic: *F_(1.45, 263.09)_ =* 57.76*, p* < 0.001; t-test between Information and Aesthetic options: *t*_267.39_=7.53, *p*<0.001; t-test between Information and Pseudo-Instrumental options: *t*_251.9_=7.81, *p*<0.001).


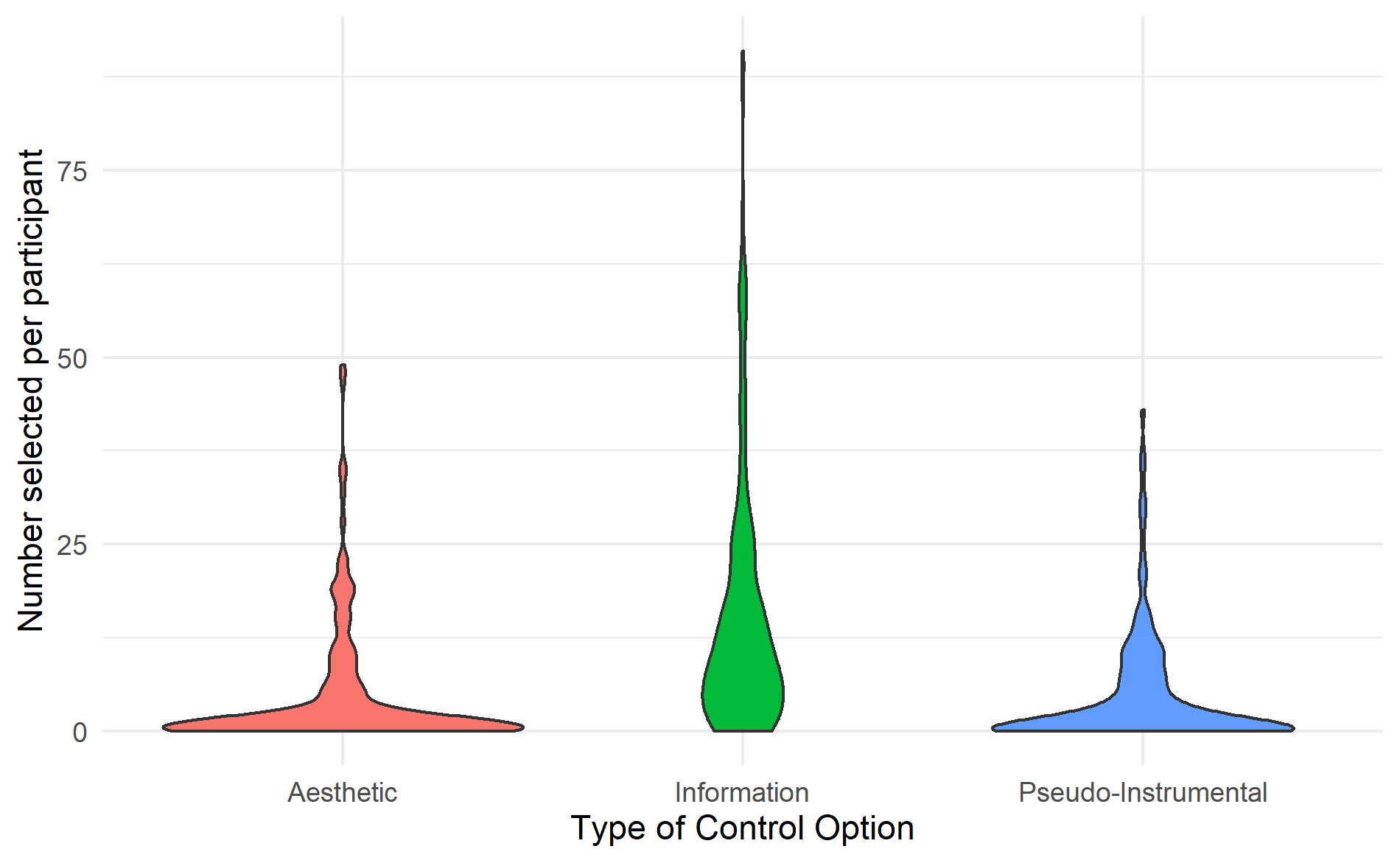


Supplementary Figure 1: Number of times each participant selected each type of control option.

## H5: Change in Control Option selection over time

We found a significant effect of block number on Control Option selection: as the task progressed, participants selected fewer Control Options (main effect of condition position in a mixed-effects model including within subject Control Option selection per block and block number, and a random effect of participant, *β* = -1.42, *p* < 0.01; Supplementary Figure 2). However, there was no main effect of including OCI-R score in this mixed model (*β*=0.058, *p*=0.245), nor an interaction effect between OCI-R score and block number (*β*=0.004, *p*=0.762).


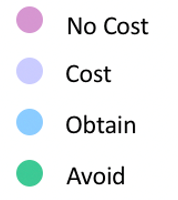

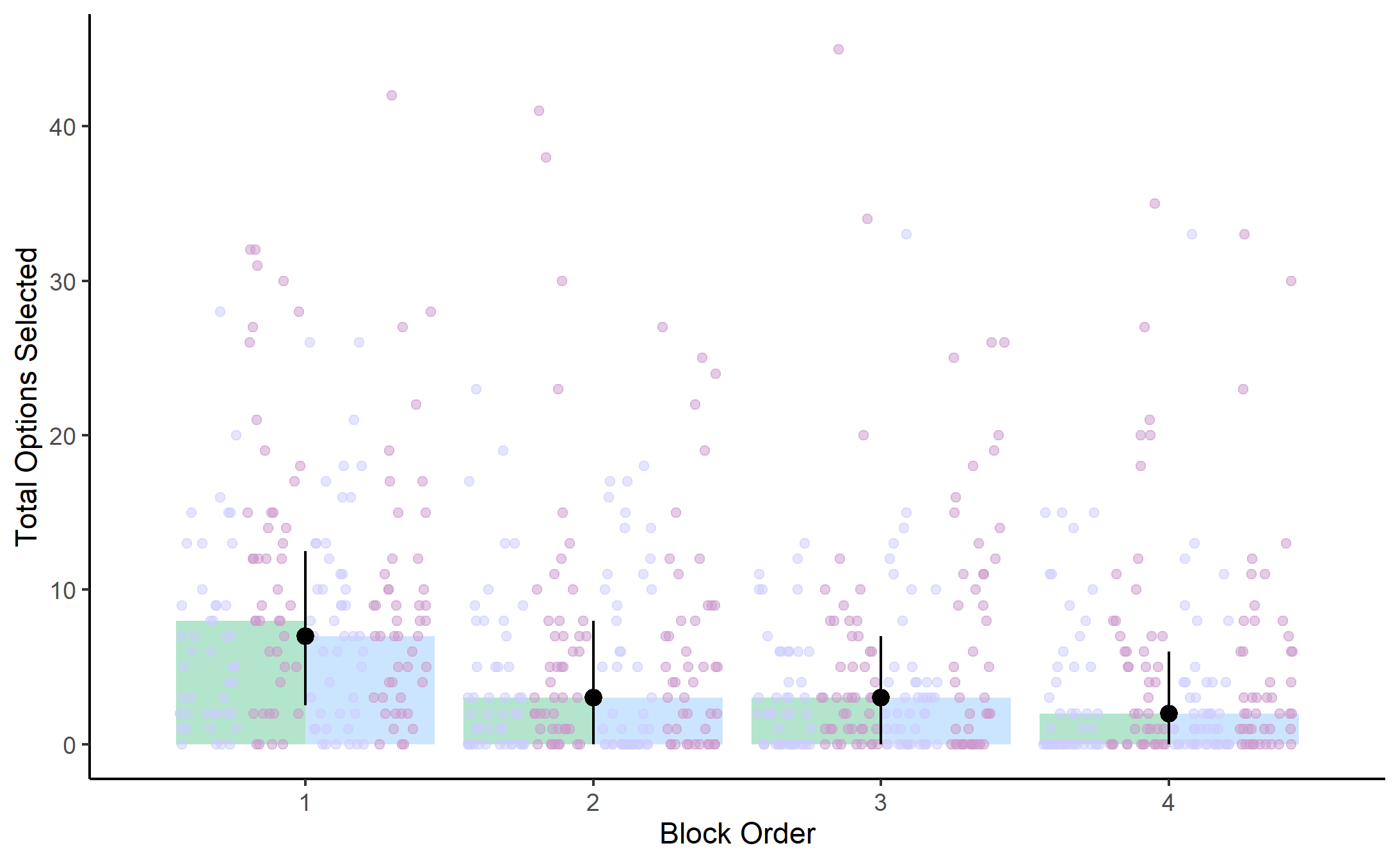


Supplementary Figure 2: Bar and point graphs showing the effect of block order on option selection. As time increases (here, we use block order as a proxy, such that ‘1’ refers to the first block participants complete), participants select fewer control options. The different conditions are shown by colour: blue and green refer to obtain particular dice numbers and avoiding particular dice numbers, and magenta and lilac refer to the no-cost and cost conditions of the experiment, respectively.

## H6: Flexible vs. fixed selection of Control Options

To assess whether participants used a flexible or fixed strategy when selecting Control Options, we calculated a repetition value for each participant by summing the number of repeat Control Option selections and dividing this by the total number of Control Options selected. We found no evidence of a relationship in either direction between this value and either total OCI-R or total BIS score in a multiple regression (OCI-R: *β*=-0.002, *p*=0.182; BIS-11: *β*=0.001, *p*=0.741).

## Structural Equation Modelling

**H1: No relationship between eating disorder symptoms and Control Option selection**

Structural Equation Modelling further confirmed our measure of IU did not mediate any relationship between Control Option selection and EAT-26 score (in neither of our compared structural equation models was there a significant regression with the latent variable of control-seeking, *p*s>0.1, and the model in which intolerance of uncertainty did not mediate the relationship was a better fit, but not significantly so: *χ^2^*(1)=2.36, *p*=0.125; BIC difference = 3, AIC difference=0. Notably, however, in this SEM IUS and EAT-26 scores were strongly related: *β*= 0.425 [SE=0.06], *p*<0.001. Supplementary figure 3.


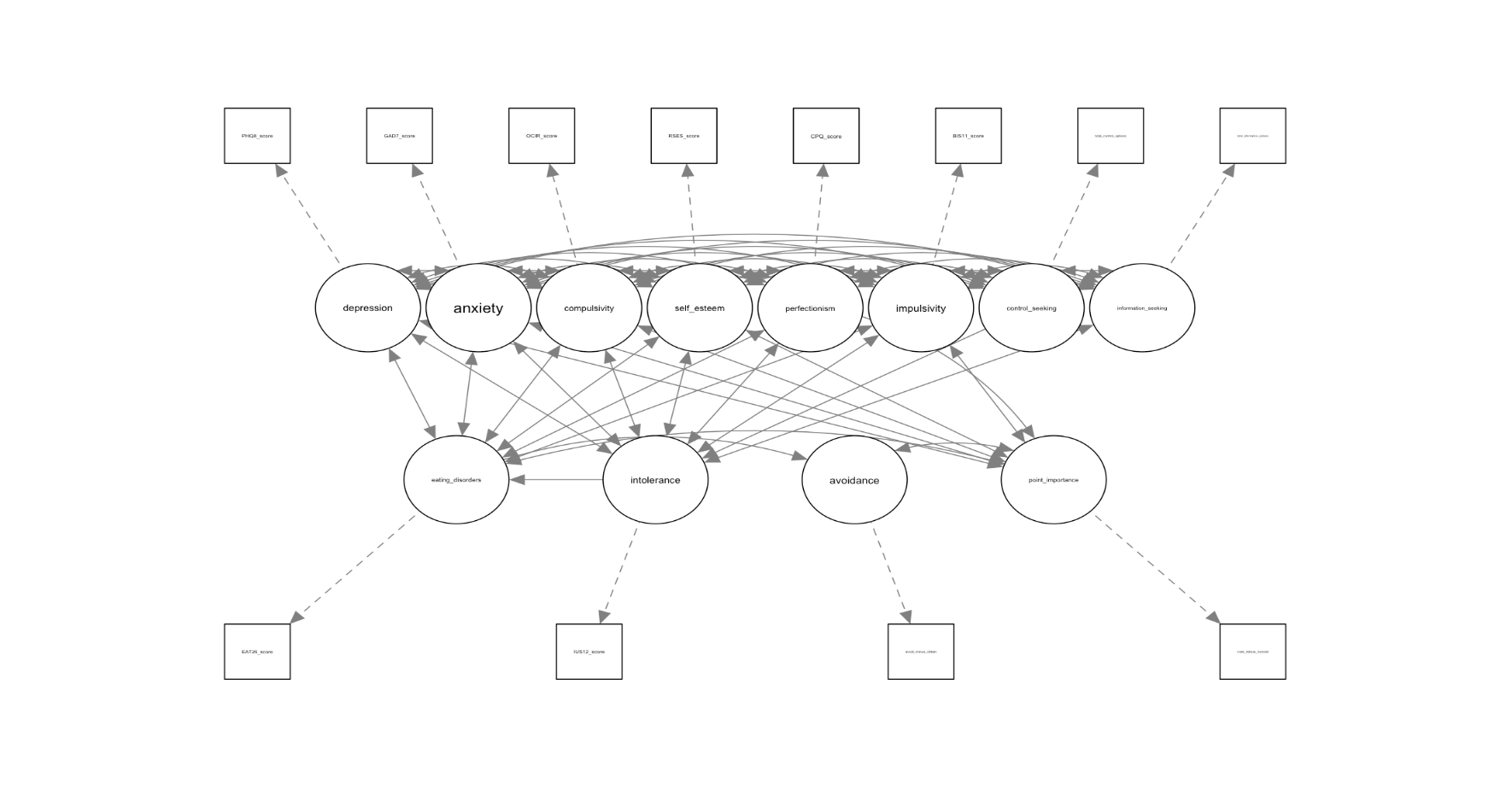


*Supplementary Figure 3: Path diagram with mediation.*

**H2: No relationship between the effect of ‘Avoid’ condition on Control Option selection**

As shown in the main manuscript, we found no significant correlation between Avoid framing and Control Option selection, this was also reflected in the structural equation model (β=-0.002 [SE=0.074], p=0.974).

**H3: No relationship between the effect of ‘Cost’ condition on Control Option selection and self-reported perfectionism**

As shown in the main manuscript we found no significant relationship between the different in Control Option selection in Cost vs No-Cost conditions and CPQ score using Spearman’s rank correlations, this was also not significant in an SEM (p>0.2).

**Sensitivity Analysis**

In the main manuscript we performed a sensitivity analysis excluding those participants who indicated their answers to the EAT-26 may be confounded. The exclusion of these participants resulted in a significant negative correlation between Cost condition and Control Option selection with CPQ. This effect is not significant in SEM (*ps*>0.2).

**Exploratory Analysis**

Structural equation modelling finds a relationship between OCI-R and Control Option selection using a single regression between compulsivity as a latent factor and control option selection: *β*=0.155 [SE=0.072], *p*=0.030.

# Discussion

During our pre-registered exploratory analyses, we found Information Control Options to be most frequently selected, whereas Aesthetic Control Option selection correlated with OCI-R score. In addition, those with higher OCI-R scores were not more likely to persevere with Control Option selection over time and neither BIS nor OCI-R influenced whether participants pursued a flexible or fixed strategy in control-seeking. Our Structural Equation Modelling confirmed the lack of relationship between control-seeking, as operationalised by the selection of Control Options, and overall eating pathology. The strongest relationship found is between OCI-R and Control Option selection, supporting findings shown in the main manuscript.

## H4: Control Option category preference

We had hypothesised that OCI-R might correlate with Information Control Option selection given the suggested relationship between compulsivity and information-gathering (NSPN Consortium et al., 2017). We don’t find evidence of increased performance-irrelevant information gathering, which could mean that where information-seeking is elevated, the information in question has, or has the illusion of having, some functional purpose.

By allowing instrumentality over the goal, the Pseudo-Instrumental Control Option was intended to provide an indication as to whether control-seeking behaviours are proactive and goal-directed. We didn’t find evidence of any such relationship, though it is not possible to assess whether such features might be important components of control seeking in EDs, as we didn’t see control-seeking in general.

We did find OCI-R to correlate positively with the selection of Aesthetic Control Options. This option was intended to represent arbitrary control-seeking, in that changing the colours of the different dice did not interfere with the goal, nor provide any marker of progress (as per the Pseudo-Instrumental and Information Control Options). This increased selection of Control Options might be consistent with the idea of increased exploratory behaviour in OCD (Mandali et al., 2019), which, if we interpret the aforementioned finding of increased information-gathering in OCD as evidence-gathering (NSPN Consortium et al., 2017), means we could interpret these results not as overt control-seeking behaviour, but as exploring or confirming the task-space by viewing and selecting different dice. Whilst we didn’t find this same behaviour in those with higher EAT-26 scores, given the overlap between EDs and OCD (Gillan et al., 2016; Godier and Park, 2014; Pearlstein, 2002), exploring whether this is an important feature of some control-seeking behaviours seen in EDs could be of interest.

One caveat to this interpretation is that the effort required to engage with the different Control Options was not equal. The Information Control Options required a single click of a button in order to display information, whereas to change the colour of the dice multiple clicks were required. This was also the case for the Pseudo-Instrumental Control Options. In our results we see a marked difference in the incidence of Control Option selection by category, with Information Options chosen most often. So although these Control Options are conceptually different in the type of control they offer to participants, they also differ in the levels of effort required for engagement, and so Control Option selection may be measuring any number of cognitive processes pertaining to effort (Inzlicht et al., 2018).

## H5: Change in Control Option selection over time

We didn’t see any evidence of perseverance in those with higher OCI-R scores, meaning that where we do see increased Control Option selection relating to OCI-R, this is not due to maintained interaction with the task over time, but rather are due to overall levels of engagement with the task.

## H6: Flexible vs. fixed interaction with Control Options

Whilst examining patterns of Control Option selection, we didn’t find any evidence of a bias toward more flexible or more fixed response styles. However, given the overall number of Control Options selected per participant was low, we were most likely unable to detect any preferred strategy for interacting with the task.

# Conclusion

In this paradigm we begin to see control-seeking behaviours in those with higher OCI-R scores, but we don’t see control-seeking in those with disordered eating attitudes. It is unclear as to whether this type of evidence-gathering may also be present in those who score higher on the EAT-26 given the skew of scores in our sample, but this could be worth investigating as a potential behavioural overlap between OCD and EDs, or point to the role of compulsivity in control-seeking.

# Supplementary References:

Gillan, C.M., Kosinski, M., Whelan, R., Phelps, E.A., Daw, N.D., 2016. Characterizing a psychiatric symptom dimension related to deficits in goal-directed control. eLife 5. https://doi.org/10.7554/eLife.11305

Godier, L.R., Park, R.J., 2014. Compulsivity in anorexia nervosa: a transdiagnostic concept. Front. Psychol. 5. https://doi.org/10.3389/fpsyg.2014.00778

Inzlicht, M., Shenhav, A., Olivola, C.Y., 2018. The Effort Paradox: Effort Is Both Costly and Valued. Trends in Cognitive Sciences 22, 337–349. https://doi.org/10.1016/j.tics.2018.01.007

Luigjes, J., Lorenzetti, V., de Haan, S., Youssef, G.J., Murawski, C., Sjoerds, Z., van den Brink, W., Denys, D., Fontenelle, L.F., Yücel, M., 2019. Defining Compulsive Behavior. Neuropsychol Rev 29, 4–13. https://doi.org/10.1007/s11065-019-09404-9

Mandali, A., Weidacker, K., Kim, S.-G., Voon, V., 2019. The ease and sureness of a decision: evidence accumulation of conflict and uncertainty. Brain 142, 1471–1482. https://doi.org/10.1093/brain/awz013

NSPN Consortium, Hauser, T.U., Moutoussis, M., Dayan, P., Dolan, R.J., 2017. Increased decision thresholds trigger extended information gathering across the compulsivity spectrum. Translational Psychiatry 7. https://doi.org/10.1038/s41398-017-0040-3

Pearlstein, T., 2002. Eating disorders and comorbidity. Arch Womens Ment Health 4, 67–78. https://doi.org/10.1007/s007370200002
